# Pancreatic adenocarcinoma protein-protein interaction network analysis 

**Published:** 2017

**Authors:** Mostafa Rezaei-Tavirani, Sina Rezaei-Tavirani, Nayebali Ahmadi, Nosratollah Naderi, Saeed Abdi

**Affiliations:** 1 *Proteomics Research Center, Shahid Beheshti University of Medical Sciences, Tehran, Iran*; 2 *Foodborne and Waterborne Diseases Research Center, Research Institute for Gastroenterology and Liver Diseases, Shahid Beheshti University of Medical Sciences, Tehran, Iran*; 3 *Basic and Molecular Epidemiology of Gastrointestinal Disorders Research Center, Research Institute for Gastroenterology and Liver Diseases, Shahid Beheshti University of Medical Sciences, Tehran, Iran*; 4 *Gastroenterology and Liver Diseases Research Center, Research Institute for Gastroenterology and Liver Diseases, Shahid Beheshti University of Medical Sciences, Tehran, Iran*

**Keywords:** pancreatic adenocarcinoma, Protein-Protein Interaction, biomarker panel, gene ontology, cluster

## Abstract

**Aim:**

Gene assessment of pancreatic adenocarcinoma disease via protein-protein interaction (PPI) Network Analysis.

**Background:**

Diagnosis, especially early detection of pancreatic adenocarcinoma as a lethal disease implies more investigation. PPI Network Analysis is a suitable tool to discover new aspects of molecular mechanism of diseases.

**Methods:**

In the present study the related genes to pancreatic adenocarcinoma are studied in the interactome unit and the key genes are highlighted. The significant clusters were introduced by Cluster-ONE application of Cytoscape software 3.4.0. The genes are retrieved from STRING date base and analyzed by Cytoscape software. The crucial genes based on analysis of central parameters were determined and enriched by ClueGO v2.3.5 via gene ontology.

**Results:**

The number of 24 key genes among 794 initial genes were highlighted as crucial nodes in relationship with pancreatic adenocarcinoma. All of the key genes were organized in a cluster including 216 nodes. The main related pathways and cancer diseases were determined.

**Conclusion:**

It was concluded that the introduced 24 genes are possible biomarker panel of pancreatic adenocarcinoma.

## Introduction

 Cancer is one of the most causes of deaths in the world. One of the most common cancer is pancreatic adenocarcinoma. Pancreatic cancer was ranked the fifth leading death of cancer in the world (1). Since late detection of pancreatic adenocarcinoma is accompanied with catastrophic situation, it is tried to find the new biomarker panel related to effective prognosis of pancreatic cancer (2). It is reported that seven miRNAs expression level in pancreatic cancer is altered (3). PPI network analysis to discover new biomarkers and assessment of different diseases, have attracted great attention of scientists (4). In this approach many genes, proteins, metabolites or RNAs which are related to a certain disease are collected and organized in an interactive organization as interactome (5-7). Each element (the node) plays an especial role in the network. In the scale free type of the networks a few number of the nodes which are characterized as central nodes play crucial roles such as control of the other nodes and facilitating speed of information circulation between the elements of the network (8, 9). The important features of central nodes are hub-nodes, bottleneck nodes and high value closeness nodes (10). The key nodes of the constructed network can be introduced as the informative biomarker panel (11). There are some dense parts (clusters) in a network that the nodes of these regions are interacted closely so they control similar pathways. The clusters form cores in center of the networks (12). Gene ontology (GO) that exposes molecular function, cellular components, biological processes and the biological pathways can be used as a useful tool to analysis of the role of a gene in biological environment (13). In the present study, pancreatic adenocarcinoma PPI network is constructed by Cytoscape software by using the genes of STRING data base. The candidate biomarker panel and the related biological pathways are introduced. 

**Table 1.** The 24 crucial nodes related to the PPI network of pancreatic adenocarcinoma are presented. D, BC and CC are abbreviations of degree, betweenness centrality and closeness centrality respectively

**Name**	**Description**	**D**	**BC**	**CC**
TP53	tumor protein p53	301	1	0.63
GAPDH	glyceraldehyde-3-phosphate dehydrogenase	266	0.29	0.61
VEGFA	vascular endothelial growth factor A	253	0.29	0.59
AKT1	v-akt murine thymoma viral oncogene homolog 1	250	0.29	0.59
ALB	albumin	249	0.43	0.59
EGFR	epidermal growth factor receptor	239	0.29	0.59
MYC	v-myc myelocytomatosis viral oncogene homolog (avian)	232	0.29	0.58
EGF	epidermal growth factor	230	0.14	0.58
INS	Insulin	218	0.29	0.57
PIK3CA	phosphatidylinositol-4,5-bisphosphate 3-kinase, catalytic subunit alpha	212	0.14	0.57
JUN	jun proto-oncogene	203	0.07	0.56
IL6	interleukin 6 (interferon, beta 2)	201	0.14	0.55
SRC	v-src sarcoma (Schmidt-Ruppin A-2) viral oncogene homolog (avian)	199	0.29	0.56
HRAS	v-Ha-ras Harvey rat sarcoma viral oncogene homolog	192	0.14	0.55
MAPK1	mitogen-activated protein kinase 1	190	0.14	0.56
CCND1	cyclin D1	190	0.14	0.55
CDH1	cadherin 1, type 1, E-cadherin (epithelial)	184	0.14	0.55
TNF	tumor necrosis factor	184	0.07	0.55
MAPK3	mitogen-activated protein kinase 3	182	0.07	0.55
IGF1	insulin-like growth factor 1 (somatomedin C)	176	0.07	0.54
PTEN	phosphatase and tensin homolog	175	0.07	0.54
CTNNB1	catenin (cadherin-associated protein), beta 1, 88kDa	173	0.14	0.54
NOTCH1	notch 1	172	0.14	0.54
MTOR	mechanistic target of rapamycin (serine/threonine kinase)	155	0.07	0.54

**Figure 1.**
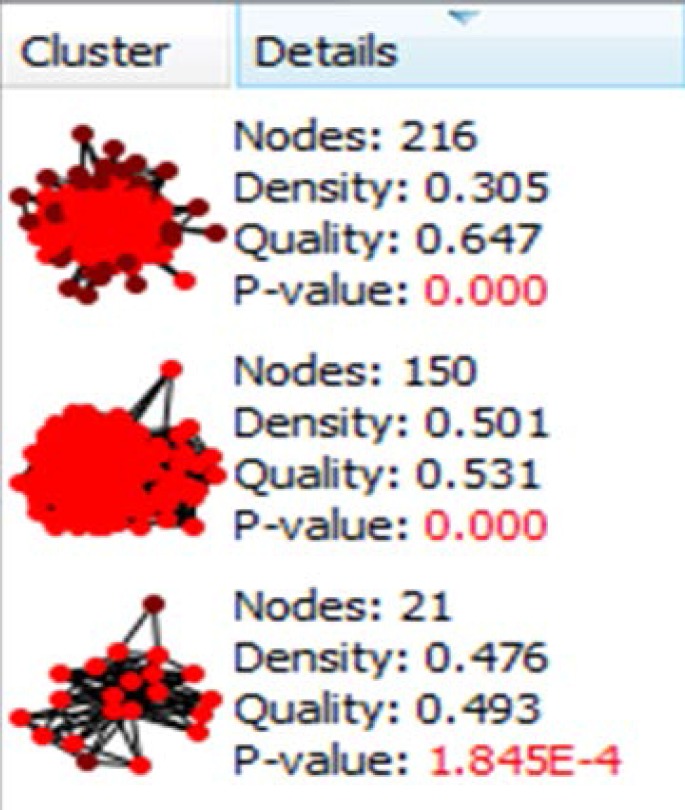
The three significant clusters related to the PPI network of pancreatic adenocarcinoma and their properties are presented

**Figure 2:**
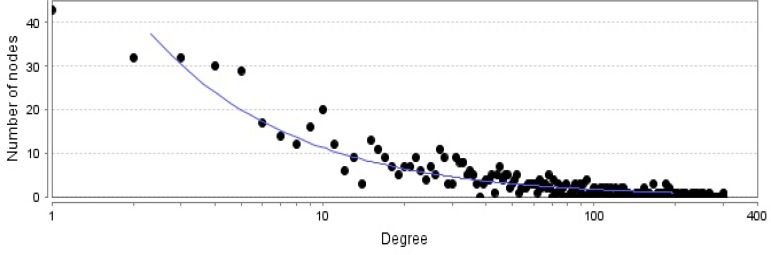
Degree distribution curve of PPI network of pancreatic adenocarcinoma is illustrated. The statistical parameters are determined as: fitted power law; y=74.353x^-0.816^, correlation; 0.910 and R-square on logarithmized value; 0.804

**Figure 3:**
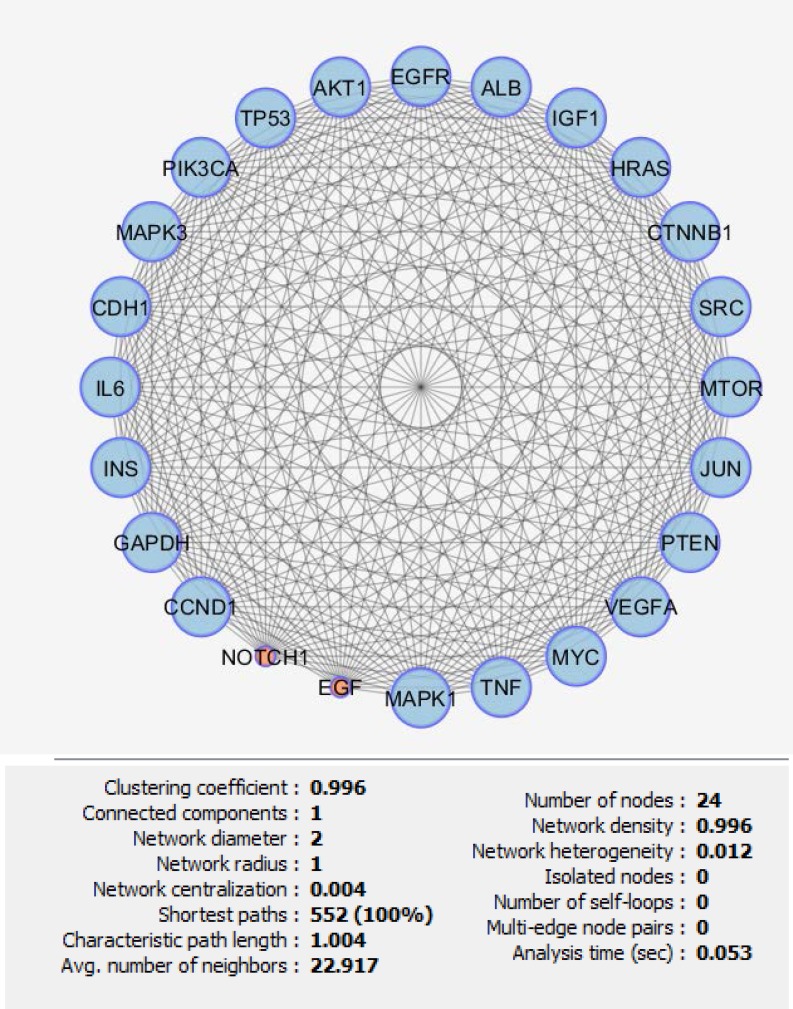
The sub network including 24 crucial nodes of pancreatic PPI network is represented. All of these genes are included in cluster-1.

**Figure 4:**
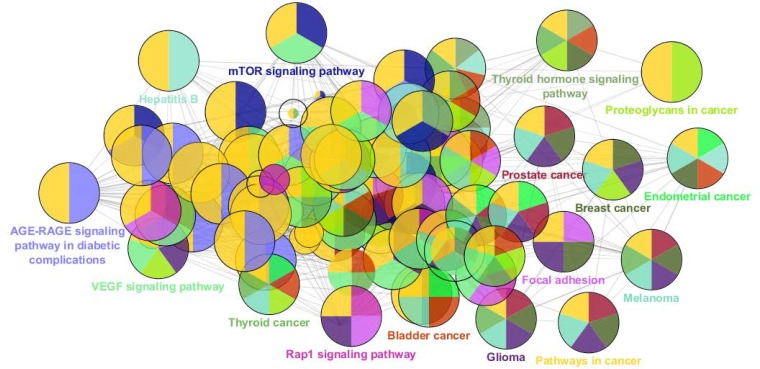
The biological pathways related to the 24 crucial nodes of pancreatic adenocarcinoma PPI network are extracted from KEGG_01.03.2017:7194. The details and statistical parameters are presented as: final kappa score groups = 60, final group size after merging: 16, GO terms: 85, GO term connections: 471. The network was constructed by ClueGO v2.3.5. The main pathways are represented but the associated pathways are hidden. The colors are corresponded on the pathways or diseases, For example the “yellow color” refers to “pathways in cancer” and “purple color” to glioma

## Methods

The significant related proteins to pancreatic adenocarcinoma are extracted from String data base (14). The 794 proteins correlated to pancreatic adenocarcinoma were found by disease query of STRING data base. This data base is one of the Cytoscape software 3.4.0 applications that provides interaction information from three different panels including disease query, protein query and PubMed query. The strength of protein interaction can be fitted for the network construction. The proteins were interacted via undirected edges and appear as an interctome unit by cytoscape software. The network was analyzed considering centrality parameters. The most important central parameter is degree value and the high value degree node is called hub-node. The hub-nodes were selected based on degree value more than mean+2SD (15). The numbers of 37 nodes were determined as hub-nodes. Two important central parameters are betweenness and closeness centralities. The 5% top nodes based on betweenness (16) and closeness values were selected as bottleneck nodes and high value closeness nodes. The common nodes between the selected three groups were identified as crucial nodes. The significant clusters (P-value≤0.001) of the network were determined by Cluster-ONE application of Cytoscape software (9). The elements of the main cluster enriched via gene ontology by ClueGO v2.3.5 plugin of Cytoscape (17). Chemical pathways were extracted from KEGG. 

## Results

There are certain genes related to diseases in STRING database. The number of 794 genes were retried for pancreatic adenocarcinoma. The constructed network was included 145 isolated and one paired nodes and a main connected component specified with 647 nodes. Network density and cluster coefficient were 0.040 and 0.397 respectively. It is corresponded to a relatively uncondensed network. However, cluster analysis showed that there are three significant clusters in the network (see figure 1). The network was analyzed based on centrality parameters. The crucial nodes (number of 24 key genes) as are described in material and methods, were determined and accompanied with their centrality parameters such as degree, betweenness centrality and closeness centrality tabulated in table 1. Surprisingly, all off the 24 important nodes are presented in cluster-1 and cluster-2. As it is depicted in figure 1 these clusters include 216 and 150 nodes respectively. It seems that these clusters are the main functional and structural parts of the network and cluster 2 are like a subcluster of cluster-1. In the other hand, presence of the 24 crucial node in the cluster-1 indicates that these nodes play main role in controlling of the network. The degree distribution curve (see figure 2) is corresponded to scale free network. In this type of the networks the most nodes have low amounts of degree and just a few nodes are characterized with high values of degree. Since the 24 introduced key genes are the main elements in the network, their connections as a subinteractome is shown in figure 3 and the related biological pathways were assessed via gene ontology enrichment analysis (see figure 4).

## Discussion

Analysis of different diseases has attracted attention of researchers and scientists in the biology and medicine fields. Some gastrohepato diseases are targeted by the experts in bioinformatics and medical informatics (18, 19). There are several molecular investigations especially genetic approaches about pancreatic adenocarcinoma. However, there is a report about switching angiogenic to pancreatic cancer but there is no complete gene analysis about pancreatic adenocarcinoma (20, 21). In current research the scale free PPI network of pancreatic adenocarcinoma is constructed and was analyzed.

 The number of 24 crucial nodes which are organized in dense part of the network were introduced as the key elements of the network. Presence of all the key nodes in the 2 significant clusters indicates that the crucial nodes are selected in a right method. In the other hand, the compressed linkages between the key nodes (as depicted in figure 3) are corresponded to interactive role of each one to control the network integrity. TP53, AKT1, EGFR, EGF, MYC and HRAS are the six well known genes that their expression level alterations in various cancers are reported frequently. There are some evidence that indicates the coloration between these genes expression changes and hepatogastro diseases (22, 23). Cowgill et al. reported mutations in TP53, KRAS, SMAD4 (DPC4) and P16 (CDKN2) genes in pancreatic cancer patients (24). 

Expression change of ALB in several cancers is highlighted. However, ALB is a blood carrier which mostly is involved in molecular transport (25). C-reactive protein/ALB ratio was used as a pancreatic cancer index by Haruki et al. (26). The relationship between GAPDH and colorectal cancers (CRCs) is investigated in the mutant cells which are attributed to CRCs. In the mutant cells GAPDH was affected ;however, the normal cells was not (27). VEGFA mediated inhibition of several cytokines in the cultured pancreatic cancer cell line is discussed in details (28).

Insulin, the main product of pancreas is well known hormone with central role in body metabolism. Insulin expression change is main feature of diabetes (29). There is a potent relationship between pancreatic ductal adenocarcinoma and diabetes. This disease is the most common and lethal feature of pancreatic cancer (30). 

It is reported that PIK3CA mutations can lead to pancreatic tumor beginning (31). The role of JUN in cell proliferation is confirmed (32). Tessari et al. were shown that the proto-oncogene C-Jun staining is a suitable approach in pancreatic cancer study (33). Rezaei-Tavirani et al. were shown TP53, EGFR, AKT1 and CTNNB1 are the main part of gastric adenocarcinoma biomarker panel (34). The effective role of CCND1 and MYC in 31 pancreatic cancer cell lines are assessed and emphasized (35). 

 Elevation of serum level of cytokines TNF and Il6 in pancreatic cancer patients were assessed by Falconer et al. (36). Since SRC is a suitable target in chemotherapy, its overexpression in pancreatic cancer refers to the crucial role of SRC in patients (37). Investigation showed that MAPK1 expression reduces in primary stage of disease (38). 

Assessment of CDH1 together 11 genes in 15 types of cancer including pancreas cancer was corresponded to effect on several biological processes such as apoptosis, cell cycle regulation (39). The finding indicates that loss of ErbB2 leads to decrement of MAPK activation of the cells of pancreatic adenocarcinoma patients (40). The previous studies indicate that loss of PTEN elevates cell proliferation and cell invasion in pancreatic adenocarcinoma. In this pathway PTEN increases Phospho-AKT and Phospho-mTOR (41). 

The IGF-1/PI3K/PTEN/Akt/NF-кB cascade was introduced as a main process in five pancreatic cancer cell line which was regulated by PTEN (42). Inhibitory role of NOTCH1 on pancreatic cancer cell invasion is reported by Wang et al. (43). 

However, the positive pieces of evidence are corresponded to the role of all 24 introduced genes in pancreatic cancer but there are some documents about relationship between these markers and the other diseases especially different types of cancers. For example expression change of EGFR in lung cancer and EGF and TP53 in gliosarcoma is reported (44-46). 

In the report of Bloomston et al. 25 microRNAs related to pancreas cancer are introduced which 21 numbers of them were overexpressed (47). It is a useful approach if the target genes of the detected microRNAs be analyzed. 

The highlighted pathways and the related diseases in figure 4 are a clear schema of relationship between these genes and the various types of cancers. Thyroid, prostate, bladder, breast and endometrial cancers accompanied with melanoma and glioma are presented in the figure 4. Yellow color which refers to “pathways in cancers” is seen in almost all of the represented circles. The significant pathways including VEGF signaling pathway, MTOR signaling pathway and Rap1 signaling pathway are attributed to the 24 crucial genes. VEGFA is the gene of row-3 in table 1. It can be proposed that a combination of a few number of these genes in a feature of a suitable biomarker panel is a useful tool in diagnosis and prognosis of pancreatic adenocarcinoma. 

In conclusion 24 genes were introduced as possible biomarker panel related to pancreatic adenocarcinoma. However, each one of them separately or in combination (68) with the other are related to the other cancers, more investigation in the field can lead to representation of a suitable biomarker panel among them.
